# Silent Killer in the Liver: A Rare Case of Primary Hepatic Angiosarcoma Presenting With Acute Liver Failure

**DOI:** 10.7759/cureus.86875

**Published:** 2025-06-27

**Authors:** Ahmed Ali Aziz, Muhammad Ali Aziz, Rida Zahid, Muhammad Amir, Rehan Shah, Ijlal Akbar Ali

**Affiliations:** 1 Internal Medicine, INTEGRIS Health Baptist Medical Center, Oklahoma City, USA; 2 Internal Medicine, University of Kentucky College of Medicine, Lexington, USA; 3 Internal Medicine, Oklahoma Heart Hospital, Oklahoma City, USA; 4 Transplant Hepatology, INTEGRIS Health Baptist Medical Center, Oklahoma City, USA; 5 Internal Medicine, Bayonne Medical Center, Bayonne, USA; 6 Internal Medicine, Section of Digestive Diseases and Nutrition, University of Oklahoma Health Sciences Center, Oklahoma City, USA

**Keywords:** chemotherapy, hepatology, liver cancer, liver transplant, oncology, primary hepatic angiosarcoma

## Abstract

Primary hepatic angiosarcoma (PHA) is a rare malignant tumor of the liver with an extremely poor prognosis even after diagnosis and treatment. It is difficult to diagnose due to a lack of disease-specific clinical features, tumor markers, or imaging findings. Due to its rarity of occurrence, treatment protocols are not yet clear. We present a rare case of PHA in a 39-year-old female who presented with right upper quadrant abdominal pain and was found to have acute liver failure and a non-resectable liver mass. She underwent a liver transplant (LT) as PHA could not be diagnosed before the transplant. She received chemotherapy and had done well for 12 months until her carcinoma relapsed. She underwent a repeat cycle of chemotherapy; however, her tumor continued to progress and is now undergoing a trial of immunotherapy.

## Introduction

Primary hepatic angiosarcoma (PHA) is a rare liver tumor originating from hepatic endothelial cells [1]. It accounts for 2% of primary malignant liver tumors and is caused by the invasive growth of vascular or lymphatic endothelial cells. It is the third most common primary hepatic tumor [1]. It has an extremely poor prognosis, and most patients survive only six to seven months; very few patients survive for more than two years after diagnosis [2]. The exact etiology of PHA remains unclear; however, its occurrence has been associated with exposure to toxins, such as vinyl chloride, arsenic, anabolic steroids, exogenous estrogens, and radiation. The etiology remains undetermined in 75% of the cases [1,3]. PHA is difficult to diagnose due to a lack of specific tumor markers or specific imaging findings [4]. Imaging studies might help in the diagnosis of PHA, but in many cases it is not possible to differentiate PHA on imaging from other liver tumors that demonstrate hypervascularity, such as hemangioendothelioma, hemangiomas, or carcinomas [5,6]. Definitive diagnosis can be established by liver biopsy; however, in many cases, biopsy is inconclusive [7]. Treatment options are limited due to the rarity of the disease and limited literature available on management [8]. We present a rare case of a 39-year-old patient who presented with right upper quadrant abdominal pain, and workup showed non-resectable hepatic lesions. Her hospital course was complicated by acute liver failure. Her liver biopsy, performed twice, was inconclusive, and hence she underwent a liver transplant (LT). Biopsy of her explanted liver revealed PHA. She underwent chemotherapy and remained tumor-free for 12 months until her PHA relapsed after 12 months. She failed a trial of repeat chemotherapy and is now being considered for immunotherapy.

## Case presentation

**Figure 1:**
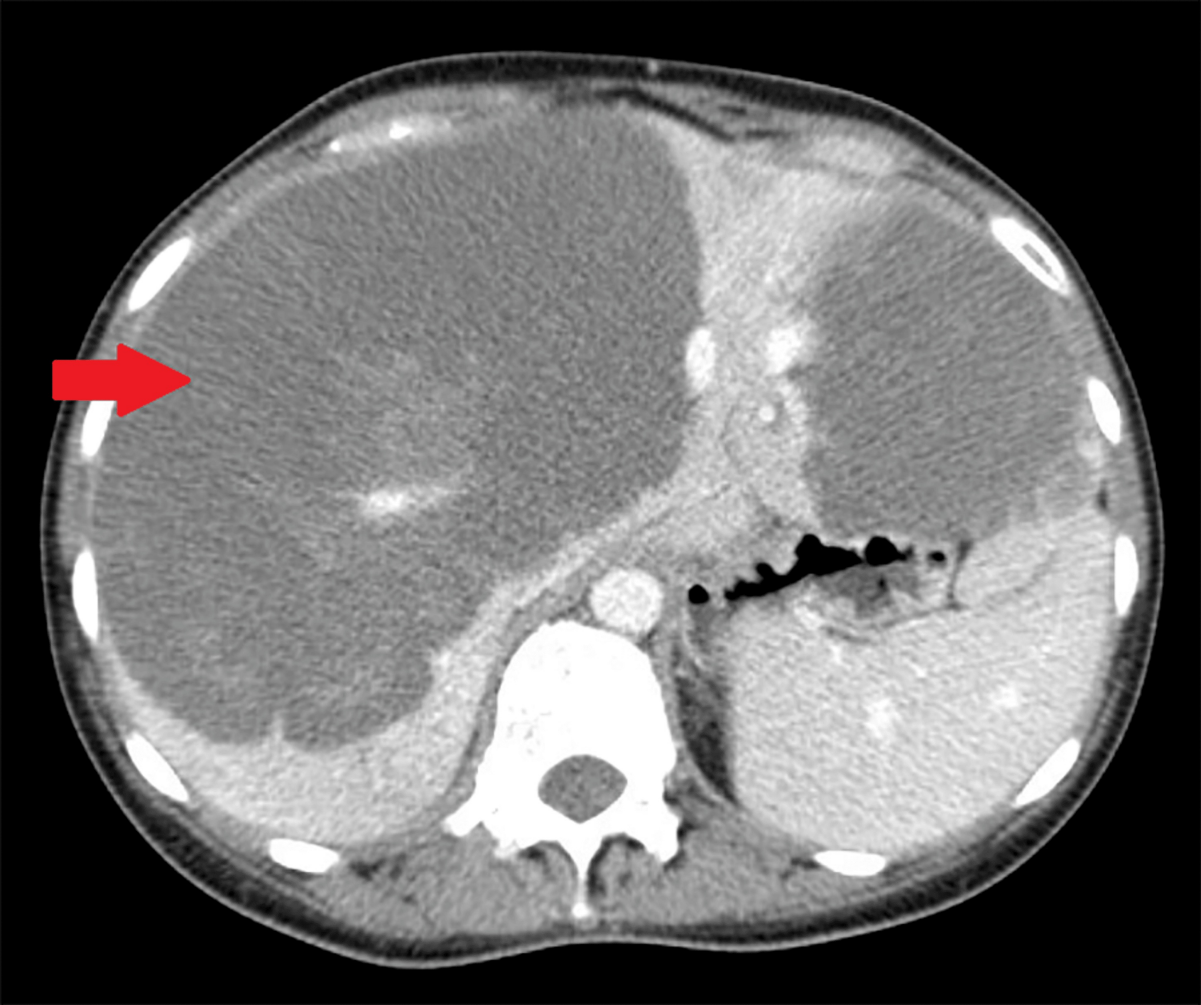
Transverse section of computed tomography (CT) scan of the abdomen and pelvis. The arrow points towards the liver mass.

A 39-year-old woman with a history of iron deficiency anemia presented to our emergency department for evaluation of right upper abdominal pain ongoing over the last two weeks. Her abdominal pain was not related to food intake, and she denied any nausea, vomiting, diarrhea, constipation, hematochezia, or melena. She had an outpatient computed tomography (CT) scan of the abdomen and pelvis two weeks prior to presentation for abdominal pain, which showed hepatomegaly with multiple hepatic lesions, the largest measuring up to 17 cm (Figure 1).

She was planned for an outpatient biopsy, but had worsening abdominal pain, prompting her to come to the ER for evaluation. She denied any history of liver disease, family history of malignancy, use of recreational drugs, herbal supplements, and hormone replacement pills. She reported occasional alcohol intake on weekends with no binge drinking. On presentation, she was vitally stable. Her physical examination showed non-distended abdomen with minimal tenderness to palpation in the right upper quadrant and hepatomegaly. Her lab work was significant for aspartate transaminase (AST) 44 U/L, alanine transaminase (ALT) 43 U/L, alkaline phosphatase (ALP) 243 U/L, total bilirubin 1.3 mg/dL, and international normalized ratio (INR) 1.7. Hepatology service was consulted. Her blood workup for acute viral hepatitis panel, acute human immunodeficiency virus (HIV) panel, ceruloplasmin levels, anti-nuclear antibody (ANA), and anti-smooth muscle antibody was negative (Table 1).

**Table 1:** Laboratory results on initial admission. BUN: blood urea nitrogen

Lab parameters (units)	Reference range	Lab results
Sodium (mmol/L)	135-145	136
Potassium (mmol/L)	3.5-5.5	3.6
Chloride (mmol/L)	96-106	103
BUN (mg/dL)	6-24	12
Creatinine (mg/dL)	0.7-1.3	0.54
Bicarbonate (mmol/L)	22-29	24
Glucose (mg/dL)	70-100	90
Aspartate aminotransferase (U/L)	13-39	44
Alanine aminotransferase (U/L)	7-52	43
Alkaline phosphatase (U/L)	34-104	243
Total bilirubin (mg/dL)	0.2-1.3	1.3
White blood cells (×10³/µL)	3.8-10.2	4.8
Hemoglobin (g/dL)	12.9-16.7	7.0
Hematocrit (%)	39.2-48.8	22.0
Platelets (×10³/µL)	150-450	102
International normalized ratio (INR)	0.8-1.1	1.7
Partial thromboplastin time (s)	25-35	28
Prothrombin time (s)	11-13.5	16.5
Ceruloplasmin (mg/dL)	14-40	30
Hepatitis A antibody, total	Non-reactive	Non-reactive
Hepatitis B core antibody, total	Non-reactive	Non-reactive
Hepatitis C antibody, total	Non-reactive	Non-reactive
Human immunodeficiency virus (HIV) Ag/Ab	Non-reactive	Non-reactive
Anti-smooth muscle antibodies (ASMA)	Non-reactive	Non-reactive
Antinuclear antibody	Non-reactive	Non-reactive

**Figure 2:**
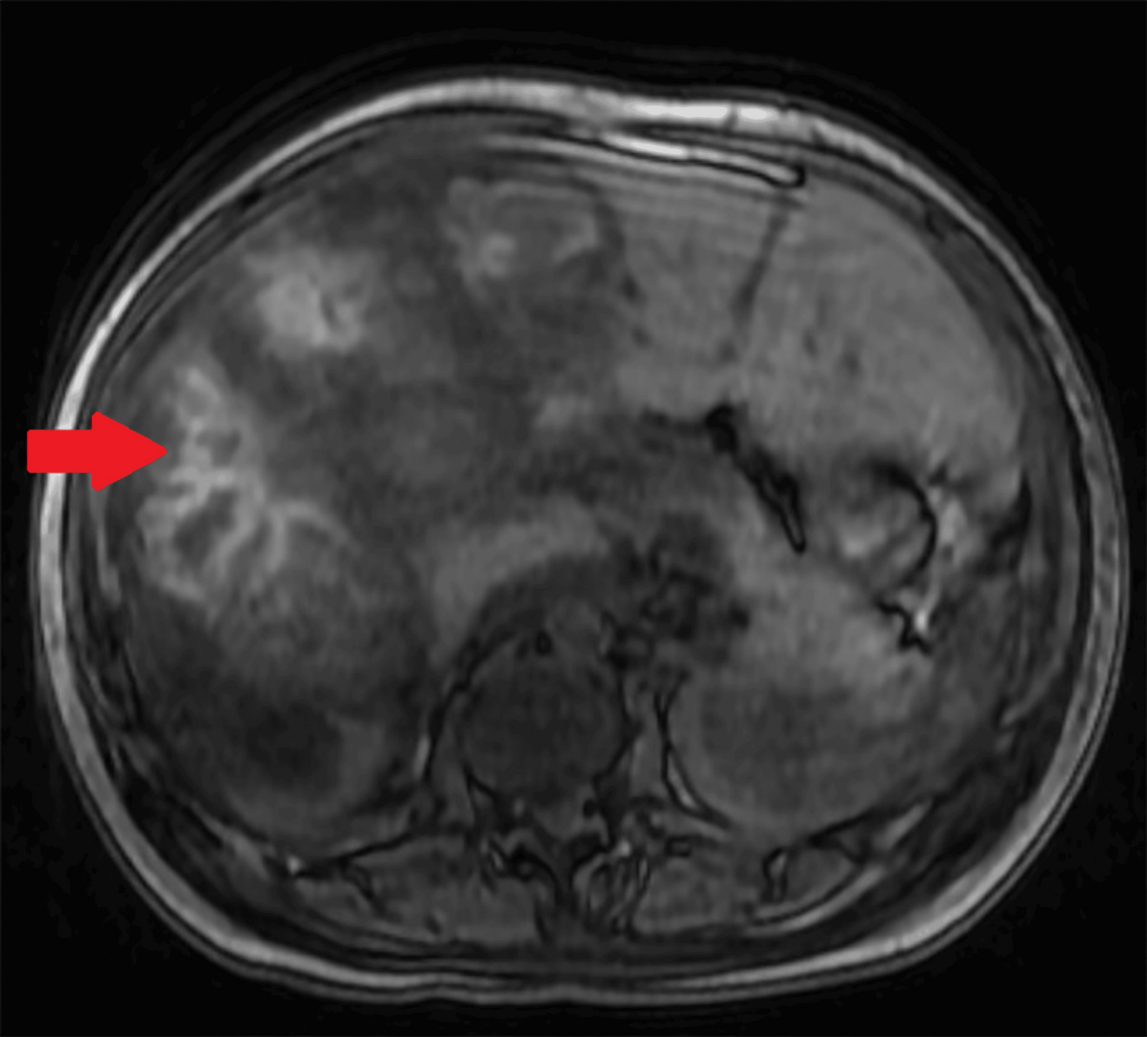
Magnetic resonance imaging (MRI) of the abdomen with and without intravenous contrast. The arrow points towards the liver mass with hemorrhage.

Her tumor markers, including alpha-fetoprotein (AFP), carcinoembryonic antigen (CEA), and carbohydrate antigen (CA) 19-9, were within normal limits. Entamoeba histolytica testing was negative. She underwent liver biopsy, which was complicated by post-procedural hemorrhage, and she required embolization of a branch of the left hepatic artery. Post-biopsy magnetic resonance imaging (MRI) of the abdomen with and without intravenous contrast was concerning for multiple small and large hepatic adenomas with hemorrhage in most of the large lesions (Figure 2).

Her hospital course was complicated by ascites on day five, and she required paracentesis. Her liver biopsy returned as non-diagnostic, containing hemorrhagic/blood clot components. Patient underwent repeat liver biopsy that again came back as inconclusive, but was thought to be either hemangioendothelioma or hemangiosarcoma as per the pathologist. She was deemed to be at excessive bleeding risk for a wedge or laparoscopic biopsy. She was not on any anabolic steroids or hormonal supplements and did not qualify for medical management for her liver lesions. Due to the diffuse nature of liver lesions, she was not considered a surgical candidate for resection of these lesions. She was thought to benefit fully from liver transplant (LT) and hence underwent a thorough work-up and was deemed stable enough to undergo transplant surgery.

**Figure 3:**
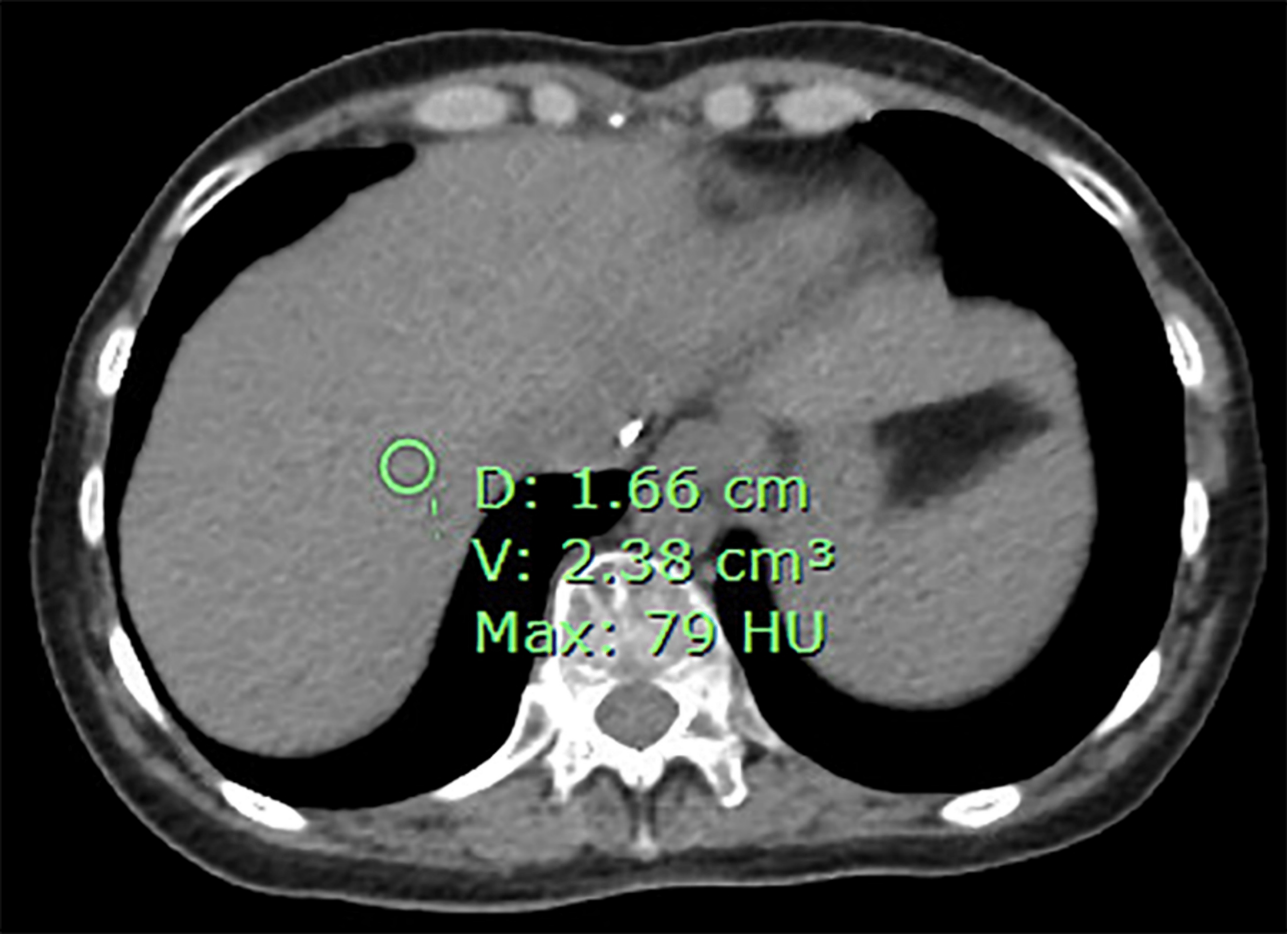
Positron emission tomography (PET) scan showing 2.1 cm hypoenhancing area in hepatic segment 8 marked by green circle. D: diameter; V: volume; Max: maximum; HU: Hounsfield units

She was discharged and returned to the hospital five weeks later for LT surgery. She was cytomegalovirus (CMV) positive while the donor was CMV negative. She tolerated the procedure well and was started on immunosuppression with mycophenolate mofetil 500 mg twice daily, prednisone tapered from 50 mg daily to 10 mg daily, tacrolimus 2 mg twice daily, and valganciclovir 450 mg daily. Pathology of her explanted liver showed angiosarcoma involving both lobes, while lymph nodes were negative for malignancy. She was seen by the oncology service to determine the need for adjuvant chemotherapy. She was started on an adjuvant chemotherapy regimen consisting of gemcitabine and docetaxel for four rounds of treatment with pegfilgrastim support. A baseline positron emission tomography (PET) scan and nuclear medicine (NM) bone scan performed prior to initiation of chemotherapy were negative for any metabolically active lesions or osseous metastasis, respectively. She completed four cycles of chemotherapy. Her post-chemotherapy PET and NM bone scans, performed approximately nine months post-transplant, were again negative for any metabolically active lesions or osseous metastasis, respectively. A Signatera molecular residual disease assay (MRD) test was performed, which was positive (0.22 mean tumor molecules {MTM}/mL). Twelve months after her LT, a screening PET scan was performed and showed a new 2.1 cm hypoenhancing area in hepatic segment 8 (Figure 3).

**Figure 4:**
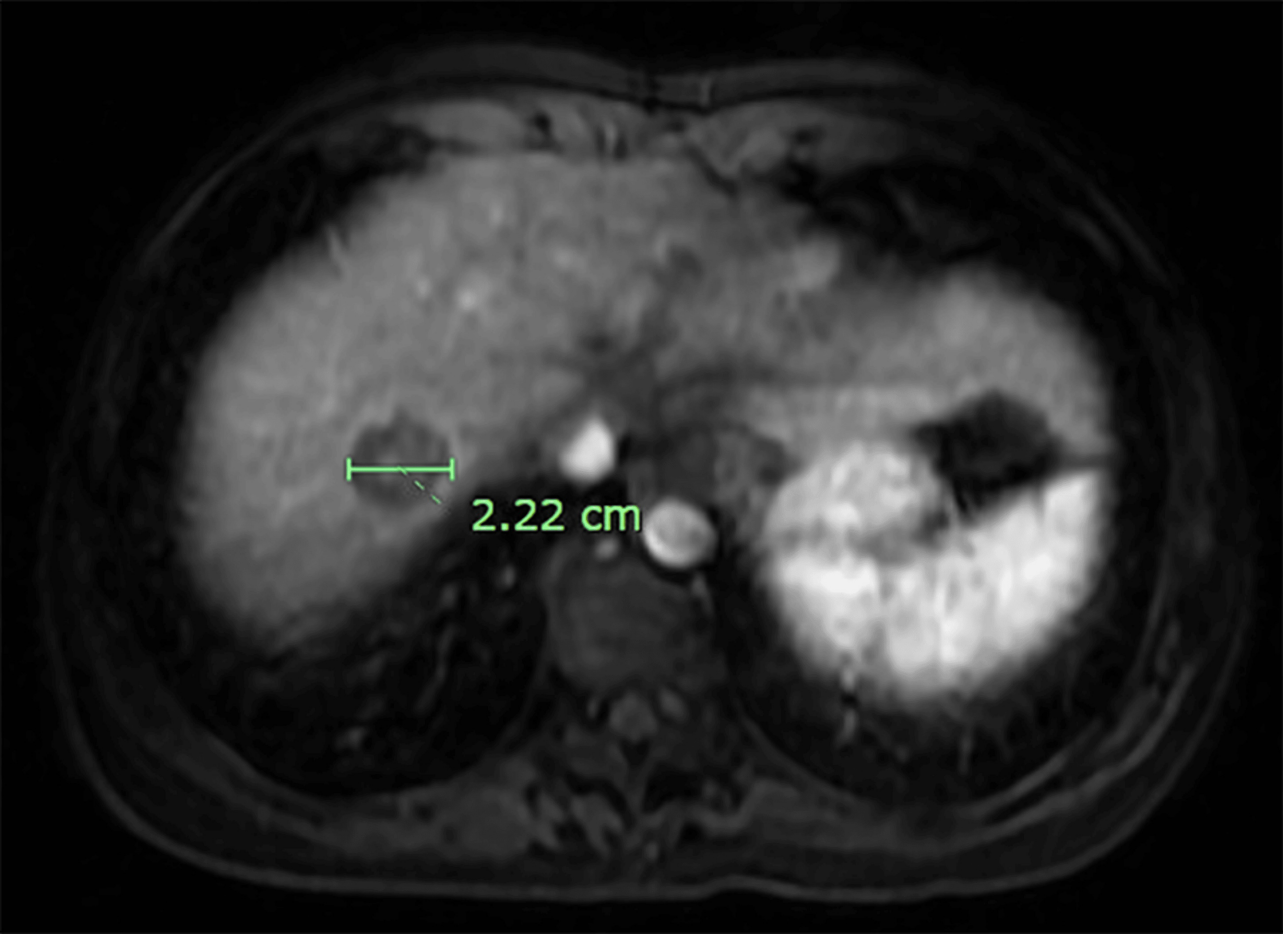
Magnetic resonance imaging (MRI) of the abdomen with and without intravenous contrast. The green line shows the maximum diameter of the liver nodule detected in hepatic segment 8.

An MRI abdomen with and without contrast was performed to further evaluate the lesion detected on the PET scan. MRI showed two non-enhancing T2 hyperintense nodules that measure up to 2.2 cm in hepatic segment 8, concerning for recurrent/metastatic disease (Figure 4).

Patient was seen by oncology and started on chemotherapy with ifosfamide and adriamycin regimen and completed four cycles. However, she again showed progressive disease on MRI, and her cancer molecular profiling indicated high microsatellite instability (MSI), high tumor mutational burden (TMB), and positive programmed death ligand-1 (PD-L1) status. She has now started on immunotherapy with pembrolizumab.

## Discussion

PHA is the most common malignant hepatic mesenchymal tumor and accounts for 2% of all primary neoplasms of the liver [1]. Exposure to carcinogenic substances, such as vinyl chloride and arsenic, is associated with increased risk of this malignancy. Many idiopathic cases have been reported too [3].

Patients usually have nonspecific clinical symptoms, such as abdominal pain, fatigue, ascites, and jaundice [1]. Tumor markers, including AFP, CA 19-9, and CEA, can be normal. PHA can present as different growth patterns on imaging. It might present as multiple nodules, a single large dominant mass, or as a combination pattern of a dominant mass with small nodules.

It is often difficult to diagnose PHA due to its low incidence rate and lack of specific clinical manifestations, laboratory findings, and due to the insufficiency of typical imaging features. Some studies suggest that the presence of non-peripheral enhancement and arteriovenous short circuit on contrast-enhanced CT is suggestive of PHA, while the absence or scarcity of peripheral nodular enhancement, arteriovenous short circuit on contrast-enhanced CT is suggestive of hepatic hemangioma [9-11]. Liver biopsy is said to be the gold standard for confirming the diagnosis of PHA [12].

The most effective treatment option for PHA is radical resection followed by targeted chemotherapy [13]. However, due to the aggressive nature and multifocal distribution within the liver parenchyma, less than 20% of patients qualify for radical resection of PHA [1]. Liver transplantation is not recommended in these patients as no survival benefit has been noticed after LT [14]. The median survival duration after liver transplantation in PHA is reported to be less than seven months [15]. Only 3% of patients survive for more than two years [3].

Our patient was 39 years old and presented with nonspecific symptoms of right upper quadrant abdominal pain. Her tumor markers were negative, and imaging was non-diagnostic for PHA. Her liver biopsy was inconclusive two times. She underwent LT because of liver failure and because she was not a candidate for medical treatment or radical resection of her liver lesions. Biopsy of her explanted liver confirmed PHA. Had her diagnosis been established before, she would not have been an LT candidate. She underwent chemotherapy with gemcitabine and docetaxel. Standard adjuvant chemotherapy regimens have not been established for PHA till now due to its scarcity of occurrence and such poor prognosis and grim life expectancy from the time of diagnosis [2]. Gemcitabine and docetaxel are commonly used for the treatment of soft tissue sarcoma [2,16]. She remained disease-free for almost 12 months until her repeat PET and MRI scans showed recurrence of her PHA. Our patient is one of the rare patients who have survived PHA for more than 12 months. Since she failed previous chemotherapy and had a recurrence of her disease, she was started on chemotherapy with ifosfamide and adriamycin. On review of literature, the combination of ifosfamide and doxorubicin has demonstrated improved survival in sarcomas as well [17]. She completed four treatment cycles. However, she again showed progressive disease on MRI, and her cancer molecular profiling indicated high microsatellite instability (MSI), high tumor mutational burden (TMB), and positive programmed death ligand-1 (PD-L1) status. Recent literature has shown PD-L1 inhibitors to be effective in treating PD-L1-positive PHA [18]. Since the patient’s tumor was PD-L1 positive, she then started immunotherapy with the PD-L1 inhibitor pembrolizumab, and her progress was monitored.

## Conclusions

PHA is a rare malignant tumor with a poor prognosis. The clinical symptoms are not typical, imaging findings are easily confused with other hepatic tumors that have high vascularity, such as hemangioendothelioma, hemangiomas, and biopsy might not always be conclusive. Treatment options are limited as the disease is very rare, and there is limited data available on treatment and management. Radical resection, if possible, followed by chemotherapy, is an effective treatment option. Liver transplantation should not be considered, as it has not been shown to improve mortality. This report is an important addition to the limited literature available on PHA and offers diagnostic and treatment information for physicians who might encounter such patients in clinical practice.
